# The complete genome sequence of *Androctonus mauritanicus,* the Moroccan black thick-tailed scorpion

**DOI:** 10.56179/001c.55548

**Published:** 2022-11-10

**Authors:** Douae El Ghoubali, Stacy Pirro, Sofia Sehli, Mohammed Merzouki, Najib Al Idrissi, Lahcen Belyamani, Salsabil Hamdi, Hassan Ghazal

**Affiliations:** 1 Laboratory of Genomics and Bioinformatics, Mohammed VI University of Health Sciences,; 2 Biodiversity, Iridian Genomes,; 3 Faculty of Sciences and Techniques, University Moulay Slimane,; 4 Department of Surgery, Mohammed VI University of Health Sciences,; 5 Mohammed VI University of Health Sciences,; 6 Department of Research, Institut Pasteur,; 7 National Center for Scientific and Technical Research

**Keywords:** scorpion, arthropoda, africa, genome, morocco

## Abstract

*Androctonus mauritanicus* is a large scorpion indigenous to North Africa. Notable for its extremely potent venom, it is responsible for several human deaths a year. We present the whole genome sequence of this species. Illumina sequencing was performed on a genetic sample from a single wild-caught individual. The reads were assembled using a *de novo* method followed by a finishing step. The raw and assembled data are publicly available via GenBank: Sequence Read Archive (SRR10738938) and Assembly (GCA_011317285).

## Introduction

*Androctonus mauritanicus* (Buthidae, Scorpiones) is found in Morocco and Mauritania. It is a large scorpion (8–10 cm) with dark coloration. It has long pincers with the tips generally lighter in color than the rest of the body. The tail is long and wide, and the aculeus is apparent although relatively thin. Adults are normally dark brown with the legs, tips of pincers, and the abdomen a lighter brown. The adult male is easily distinguished from the female by a large notch at the base of each pincer.

This species members are prolific breeders, with females producing 50–70 offspring after a 6 month gestation period. The lifespan of both males and females in captivity is 4–5 years, with sexual maturity reached after 2 years.

Venom from all species in the *Androctonus* genus is extremely potent, with several human deaths reported each year.

## Methods

A single wild-caught individual was used for this study. DNA extraction was performed using the Qiagen DNAeasy genomic extraction kit following the standard protocol for insect tissues. A paired-end sequencing library was constructed using the Illumina TruSeq kit according to the manufacturer’s instructions. The library was sequenced on an Illumina Hi-Seq platform in paired-end, 2 × 150 bp format. The resulting fastq files were trimmed of adapter/primer sequence and low-quality regions with Trimmomatic v0.33 (Bolger, Lohse, and Usadel 2014). The trimmed sequence was assembled by SPAdes v2.5 (Bankevich et al. 2012) followed by a finishing step using Zanfona (Kieras, O’Neill, and Pirro 2021).

## Data Availability

The genome assembly yielded a total sequence length of 1,083,961,664 bp with an N50 of 57 kb.
